# Translation and psychometric assessment of the mastectomy module of the BREAST-Q questionnaire for use in Nigeria

**DOI:** 10.1186/s41687-024-00692-1

**Published:** 2024-02-09

**Authors:** Olalekan Olasehinde, Kathleen A. Lynch, Debra A. Goldman, Olaide Agodirin, Chukwuma Okereke, Funmilola O. Wuraola, Israel Adeyemi Owoade, Promise Rebecca Akinmaye, Olusola Ajibade, Karin Barber, Joshua Ogunwale, Olusegun Alatise, T. Peter Kingham, Andrea Pusic, Anya Romanoff

**Affiliations:** 1 Department of Surgery, Obafemi Awolowo University, Ile-Ife, Nigeria; 2 Department of Psychiatry and Behavioral Sciences, Memorial Sloan Kettering Cancer Center, New York, NY USA; 3 Department of Epidemiology and Biostatistics, Memorial Sloan Kettering Cancer Center, New York, NY USA; 4 Department of Surgery, University of Ilorin Teaching Hospital, Ilorin, Nigeria; 5 Department of Surgery, Federal Medical Center Owo, Owo, Nigeria; 6 African Research Group for Oncology, Osun, Nigeria; 7 Department of Linguistics, Obafemi Awolowo University, Ile-Ife, Nigeria; 8 Department of African Studies and Anthropology School of History and Cultures, University of Birmingham, Birmingham, UK; 9 Department of Surgery, Memorial Sloan Kettering Cancer Center, New York, NY USA; 10 Global Cancer Disparities Initiatives, Memorial Sloan Kettering Cancer Center, New York, NY USA; 11 Division of Plastic Surgery, Brigham and Women’s Hospital, Boston, MA USA; 12 Department of Global Health and Health System Design, Icahn School of Medicine at Mount Sinai, New York, NY USA; 13 Department of Social and Behavioral Sciences, NYU School of Global Public Health, New York, NY USA

**Keywords:** Patient reported outcomes, BREAST-Q, Breast cancer, Nigeria

## Abstract

**Background:**

The majority of non-metastatic breast cancer patients in sub-Saharan Africa are recommended to have mastectomy. The impact of mastectomy on a predominantly young African patient population requires evaluation. The BREAST-Q is a validated patient-reported outcome measure of quality-of-life following breast surgery that has been translated into 30 languages-none in Africa. This study aimed to translate and assess the psychometric properties of the mastectomy module of the BREAST-Q for use in Nigeria.

**Methods:**

The BREAST-Q mastectomy module was translated from English to Yoruba and its psychometric properties assessed using best practice guidelines. Translation was performed in 4 steps: forward translation (x2), back translation, back translation review, and cognitive interviews with post-mastectomy patients. The translated BREAST-Q instrument was administered to post-mastectomy patients (n = 21) alongside the EORTC-QLQ BR23 to evaluate construct validity. Test-retest reliability was evaluated using intraclass correlation coefficients (ICC); surveys were re-administered 4 weeks apart.

**Results:**

The translation process identified English phrases not amenable to direct translation, including “*emotionally healthy*” and descriptions of pain (“*nagging,*” “*throbbing,*” “*sharp*”). Translations were amended to reflect local context and question intent. During cognitive interviews, patients provided suggestions to simplify complex phrases, e.g. “*discomfort in your breast area.*”. Internal consistency within scales was over 0.70 for psychosocial wellbeing (α = 0.84–0.87), sexual wellbeing (α = 0.98–0.99), physical wellbeing in chest (α = 0.84–0.86), and satisfaction with care (α = 0.89–0.93). ICC for test-retest reliability was moderate (0.46–0.63).

**Conclusions:**

The Yoruba version of the BREAST-Q mastectomy module presents a unique opportunity to adequately capture the experiences of Nigerian women post mastectomy. This instrument is being used in a pilot study of Nigerian patients to identify targets for intervention to improve the patient experience and compliance with breast cancer surgery.

## Background

Breast cancer is the most common cancer in women globally [1], and the leading cause of cancer deaths in Nigeria [2]. Breast cancer incidence in Nigeria is about 52 per 100,000, a significant increase over previous estimates of about 15/100,000 in the 1970s to 33/100,000 in the 1990s [3, 4]. Like many resource constrained countries, Nigeria has a high breast cancer incidence to mortality ratio, due mainly to late presentation and limited access to care [5, 6].

Unlike high income countries, the majority of Nigerian women are diagnosed in late stages [7, 8]. This, in addition to limited access to timely radiotherapy services, makes breast conserving operations uncommon. In most instances, modified radical mastectomy without reconstruction is recommended [9–11]. This comes at a significant psychosocial, physical and emotional cost given that the majority of those affected are relatively young women of childbearing age [12]. Earlier studies have identified the fear of mastectomy as one of the reasons for non-adherence to treatment among Nigerian women [13, 14]. Addressing the challenges associated with mastectomy will help to improve the quality of life of women who have received treatment and potentially make mastectomy more acceptable in the society.

There is currently no validated tool for assessing the impact of mastectomy on women who have received treatment for breast cancer in Nigeria. The BREAST-Q is one of the validated patient-reported outcome measures of health-related quality of life following breast surgery [15, 16]. The mastectomy module which assesses satisfaction following mastectomy is applicable to the Nigerian context. It contains various domains addressing different aspects of well-being, including physical, psychosocial, sexual and emotional wellbeing as well as satisfaction with care. Since the validation of the original version in English language, the BREAST-Q has been translated into 30 other languages across the globe, with none from sub-Sahara Africa [17].

The objective of this study was to translate and assess the psychometric properties of the mastectomy module of the BREAST-Q into Yoruba, which is one of the major indigenous Nigerian languages with about 50 million native speakers, predominantly in the south-western part of Nigeria [18]. The translation of the BREAST-Q tool into Yoruba language will allow for its use in a significant proportion of Nigerian patients and also provide a template for its translation into other African languages.

## Methods

This study assessed the psychometric properties of the translated version of the mastectomy module of the BREAST-Q questionnaire. This was done using an established guide for translation and cultural adaptation that follows the International Society for Pharmacoeconomics and Outcomes Research (ISPOR) best practice guidelines. The BREAST-Q consists of independently functioning scales that evaluate Quality of Life (including psychosocial well-being, sexual well-being, physical well-being of the chest, and adverse effects of radiation) and Satisfaction (including satisfaction with breasts, surgeons, the medical team, and office staff). The psychosocial well-being, sexual well-being, physical well-being of the chest, and satisfaction with breast domains are applicable in the preoperative setting, while all the domains can be used postoperatively. BREAST-Q scores are transformed onto a scale from 0 to 100, with higher scores representing better outcomes [19].

### Translation

The translation team consisted of the principal investigators (AR, OO), two forward translators (OA, JO), and one back translator (KB). Translation was performed in four steps: forward translation, back translation, back translation review, and patient interviews. The last step involved cognitive debriefing interviews with five patients to ensure quality of the final translation. Cognitive interviewing is a qualitative process which evaluates the manner in which patients understand, mentally process, and respond to the materials presented to them, with particular attention to breakdowns in this process. Cognitive interviewing is a gold-standard method for the development linguistic adaptation, and content validation of patient-facing materials. This process ensures that the content of items captures the most important aspects of the concepts of interest and that respondents understand how to complete the items, how to reference the correct recall period, the meaning of the items, and how to use the response option scales. Patient responses were summarized and coded for common themes. Building on practices from previous content validation studies, recurring themes or aspects of the measure identified as problematic, unclear or difficult to understand by ≥3 patients were revised.

### Assessment of psychometric properties

Psychometric evaluation of the translated measure entailed assessments of measure reliability, including test-retest reliability using intraclass correlation coefficient, internal consistency using Cronbach’s alpha and convergent validity using Spearman’s correlation. The final version was subsequently administered to 21 patients who were at least 6 months post mastectomy to assess its psychometric properties. The 6-month threshold was selected based on the expectation that the patients would have fully recovered from the physical effects of surgery and would also have had sufficient time to observe changes in their bodies. Women who met the inclusion criteria were purposively selected from different age categories to ensure the validity and applicability of the findings. The European Organization for Research and Treatment of Cancer Quality of life Questionnaire Breast cancer specific module (EORTC QLQ BR23 questionnaire) was also administered at the same time in order to assess construct validity of similar domains. BREAST-Q subscale scores were calculated using the BREAST-Q users guide for version 2.0 [20] Transformed scores were calculated with the Rasch method with tables provided in the scoring manual. The questionnaires were then re-administered after one month to assess the test-retest reliability of the translated questionnaire. A time frame of one month was selected based on our hypothesis that this period would be sufficient to reevaluate stability of patient responses, as clinically we would not expect to see major changes in patient symptomology during this time. Test-retest reliability was assessed with the intraclass correlation coefficient (ICC) using the Shrout-Fleiss reliability single score method [21]. Initial and retest values were displayed on scatter plots for each Breast-Q subscale. Internal consistency was measured with Cronbach’s alpha (expected >0.70). Convergent validity was assessed through the Spearman’s correlation of Breast-Q scores with the previously validated Yoruba translation of the EORTC QLQ-BR23. Relevant domains in the questionnaires were correlated (BREAST-Q psychosocial wellbeing and EORTC QLQ BR23 body image, BREAST-Q sexual wellbeing and BR23 sexual functioning, BREAST-Q physical wellbeing and BR23 arm symptoms BREAST-Q physical wellbeing and BR23 breast symptoms and BREAST-Q satisfaction and BR23 body image domains). All other analyses were performed with SAS 9.4 TS1M6 (Cary, NC).

## Results

### Forward translation

The forward translation was performed independently by two translators (AO and OO), both Professors of Yoruba language and native Yoruba speakers. Prior to the translation, the principal investigators had explained the concepts of the Q-Portfolio questionnaire to the Forward Translators to ensure that all concepts were understood.

Each Forward Translator prepared an independent translation of the Q-Portfolio questionnaire using simple and clear terminologies which are conceptually equivalent to the English version, rather than a literal translation. The two forward translators compared their translations and produced a reconciled version. In situations where certain words were translated differently, the two translators agreed on the word that most perfectly describes the concept of interest. The majority of words were easy to capture in Yoruba language. However, the various descriptions of pain that appear in the questionnaire, such as ‘throbbing’ and ‘nagging,’ were challenging to translate. Also, the word ‘radiation,’ which has no exact Yoruba equivalent, was difficult to translate. In order to preserve the conceptual meaning of the words, descriptive terms which conveyed the intended meanings were used.

### Back translation

The reconciled version of the translation from the two forward translators was back translated by a native English speaker (KB) who is fluent in Yoruba language. The Back Translator was blinded to the original English version of the instrument.

### Back translation review by the Q portfolio team

The Q-Portfolio team reviewed the back translation and made necessary recommendations, after which further revisions were made.

### Cognitive debriefing interviews with patients

After final approval of the translation was given by the Q-Portfolio team, cognitive debriefing interviews were conducted. This was aimed at determining if any instructions, items or response options needed to be re-translated to improve comprehension based on patient feedback. Five patients, all native Yoruba speakers who were at least 6 months post mastectomy participated in this process which resulted in the refinement of some of the questions. They were purposively selected to achieve a fair spread across the various age groups and menopausal status. Three were pre-menopausal women in their 40s while the other two were older, post-menopausal. Concepts such as ‘feeling emotional healthy’, ‘feeling sexually attractive in your clothes’, and ‘feeling normal’ were refined based on the participants’ suggestions during the debriefing exercise. The final version was a conceptually equivalent version in a language that is easy for patients to understand rather than a literal translation.

### Psychometric assessment of the BREAST-Q questionnaire

**Fig. 1:**
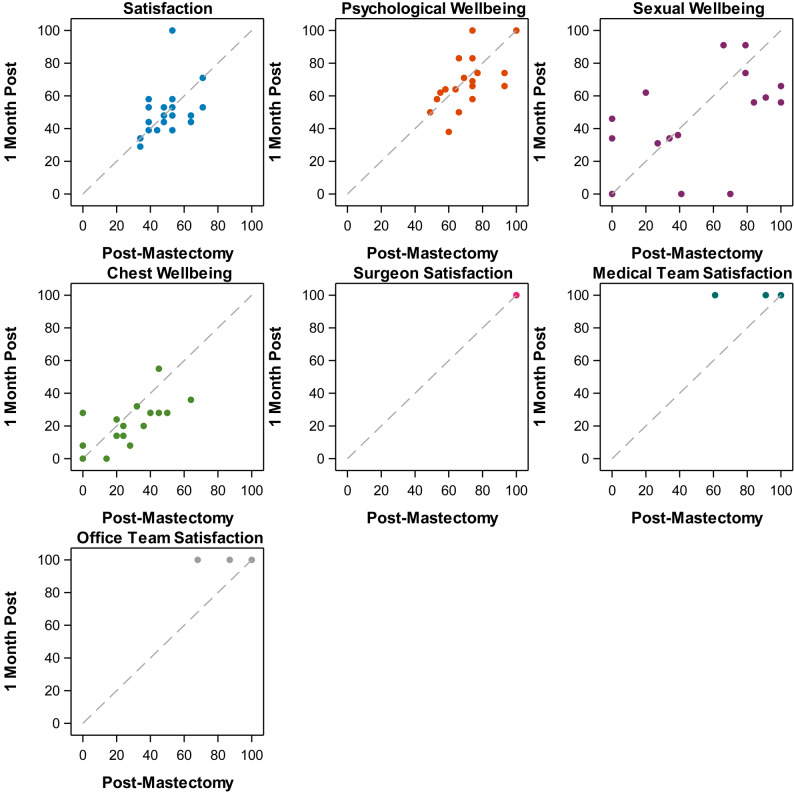
Scatterplot of Breast-Q scores

Psychometric assessment of the translated measure entailed assessment of test-retest reliability using intraclass correlation coefficient, internal consistency using Cronbach’s alpha and convergent validity using Spearman’s correlation. Overall, 21 patients participated with a period of four weeks between surveys. The median age at initial survey was 54 years (Range: 40–79). The modal age group was in the forties with nine patients (42.8%), five women (23.8%) were in their fifties and five (23.8%) were in their sixties, and two (9.5%) patients were 70 years and above. Patients completed most scales. The one exception was the sexual wellbeing domain at the initial survey time point, where only 17 of the 21 completed the scale. However, 20 of the 21 participants completed the survey at the second evaluation (Fig. 1).

### Internal consistency

Internal consistency within scales was over the 0.70 threshold for psychosocial wellbeing (α = 0.84–0.87), sexual wellbeing (α = 0.98–0.99), physical wellbeing in chest (α = 0.84–0.86), medical team satisfaction (alpha = 0.89), and office team satisfaction (α = 0.93). However, consistency for satisfaction with breasts was only moderate with α = 0.43–0.63. Satisfaction with surgeon could not be calculated due to a lack of variability (ceiling effect) (Table 1).

**Table 1:** Internal consistency between subscales

	Post-mastectomy	1 month post
	Cronbach’s alpha	Cronbach’s alpha
Satisfaction with breasts subscale score	0.43	0.63
Psychosocial wellbeing subscale score	0.84	0.87
Sexual wellbeing subscale score	0.99	0.98
Physical wellbeing-chest subscale score	0.86	0.84
Medical team satisfaction subscale score	0.89	
Office team satisfaction subscale score	0.93	

### Test-retest reliability

Reliability was moderate ranging from 0.41 (95%CI: 0.00–0.71) for the satisfaction with breasts subscale to 0.64 (95%CI: 0.30–0.84) for the physical chest wellbeing subscale. Reliability for satisfaction with surgeon, medical team, and office team could not be calculated due to a lack of variability (ceiling effect). While the reliability of the Breast-Q may be lower than the standard 0.70 threshold, these scores were slightly higher than that of the EORTC BR23, which had reliability that ranged from 0.31 (95%CI: 0.12–0.64) for arm symptoms to 0.56 (95%CI: 0.19–0.80) for breast symptoms (Tables 2 and 3).

**Table 2:** Breast-Q scores and ICC

		Post-mastectomy	1 month post	ICC [95%CI]	
Physical wellbeing-chest subscale score	Median (IQR)	24 (14–40) N = 21	17 (8–28) N = 20	0.64	[0.30–0.84]
Medical team satisfaction subscale score	Median (IQR)	100 (100–100) N = 21	100 (100–100) N = 21	NA	
Office team satisfaction subscale score	Median (IQR)	100 (100–100) N = 21	100 (100–100) N = 21	NA	
Psychosocial wellbeing subscale score	Median (IQR)	74 (60–77) N = 21	66 (58–74) N = 21	0.59	[0.24–0.81]
Satisfaction subscale score	Median (IQR)	48 (39–53) N = 21	48 (39–53) N = 21	0.41	[0.00–0.71]
Sexual wellbeing subscale score	Median (IQR)	41 (20–79) N = 17	41 (0–61) N = 20	0.56	[0.13–0.81]
Surgeon satisfaction subscale score	Median (IQR)	100 (100–100) N = 21	100 (100–100) N = 21	NA	

ICC could not be calculated for surgeon satisfaction, medical team satisfaction, and office team satisfaction due to a lack of variability

**Table 3:** QLQ-BR23-Scores and ICC

		Post-mastectomy	1 month post	ICC [95%CI]	
BR23 body image	Median (IQR)	83 (58–100) N = 21	83 (67–83) N = 21	0.44	[0.03–0.73]
BR23 systemic therapy	Median (IQR)	10 (5–14) N = 21	5 (0–10) N = 21	0.47	[0.06–0.74]
BR23 future perspective	Median (IQR)	100 (67–100) N = 21	67 (33–100) N = 21	0.49	[0.10–0.76]
BR23 breast symptoms	Median (IQR)	0 (0–17) N = 21	8 (0–8) N = 21	0.56	[0.19–0.80]
BR23 sexual functioning	Median (IQR)	33 (0–33) N = 17	33 (0–33) N = 18	0.49	[0.01–0.78]
BR23 arm symptoms	Median (IQR)	0 (0–22) N = 21	0 (0–11) N = 21	0.31	[−0.12 to 0.64]

### Correlation between BREAST-Q and EORTC-BR23

Overall, the correlations were moderate to strong. Psychosocial wellbeing and BR23 body image had ρ = of 0.56–0.68, sexual wellbeing and BR23 sexual functioning had ρ = 0.73–0.87, physical wellbeing and BR23 arm symptoms had ρ = 0.58–0.72, and physical wellbeing and BR23 breast symptoms had rho of ρ = 0.69–0.75. However, the correlation was weaker between satisfaction and BR23 body image with rho of ρ = 0.28–0.45 (Table 4).

**Table 4:** Correlation between Breast-Q and QLQ-BR23

		Post-mastectomy		1 Month Post	
		Spearman’s rho (95%CI)	*p*-value	Spearman’s rho (95%CI)	*p*-value
Satisfaction subscale score	BR23 body image	0.45 (0.02–0.74)	**0.0414**	0.28 (−0.17 to 0.63)	0.22
Psychosocial wellbeing subscale score	BR23 body image	0.68 (0.35–0.86)	**0.0005**	0.56 (0.17–0.8)	**0.0072**
Sexual wellbeing subscale score	BR23 sexual functioning	0.73 (0.34–0.9)	**0.0014**	0.87 (0.68–0.95)	**<0.0001**
Physical wellbeing-chest subscale score	BR23 arm symptoms	0.72 (0.41–0.88)	**0.0001**	0.58 (0.18–0.81)	**0.0065**
	BR23 breast symptoms	0.75 (0.47–0.89)	**<0.0001**	0.69 (0.35–0.87)	**0.0005**

*p*-values in **bold** are significant at *p* < 0.05

## Discussion

We successfully translated the mastectomy module of the BREAST-Q questionnaire into Yoruba, which is one of the major languages in Nigeria. The scientific rigour involved in the translation process as prescribed in the Q Portfolio translation guide helped ensure that the original concepts were not lost in the translation process. Overall, the process was undertaken with very minimal challenges to the measure, which were not difficult to surmount.

To date, this is the first PROM tool specifically focusing on post mastectomy related issues in any Nigerian language. Before now, the only breast cancer PROM tool tailored for use in sub-Saharan Africa was the EORTC QLQ-BR23 questionnaire, which addresses issues relating to breast cancer treatment in general [22]. The BREAST-Q delves deeply into the various aspects of patients’ physical, psychosocial and emotional wellbeing relating specifically to the effects of surgery. The need for the BREAST-Q mastectomy module is evident, given that mastectomy is the most common operation performed for breast cancer in Nigeria. The majority of affected patients being in their reproductive years makes this more imperative. A qualitative interview of a cohort of women who have had mastectomy in Nigeria had previously highlighted a variety of psychosocial and emotional challenges faced by women who have had mastectomy [12, 23]. Many of these challenges are often not addressed despite the significant impact on patients’ overall wellbeing. These issues are well captured in the BREAST-Q questionnaire, which has now been added into the Nigerian breast cancer PROM armamentarium.

Several challenges were encountered during the translation process which need to be highlighted. First was the challenge of finding a back translator. A back translator had to be an individual whose primary language is English and is fluent in Yoruba language, as defined by the translation guide—this was difficult to find. This challenge was overcome by networking with the African Languages Department of a Nigerian University which provided the link to KB, a Professor of African Cultural Anthropology who had previously worked in Nigeria. We intend adopting the same approach when translating the tool into other Nigerian languages. During the forward translation, there were challenges finding suitable translations for some of the descriptions of pain. There were no suitable words in the Yoruba dictionary to correctly translate words such as nagging, sharp, throbbing and discomfort in the context in which they were used. A similar challenge was also reported in the Danish translation of the BREAST-Q, where the authors noted that there were not enough Danish words to capture all of the English expressions [24]. To address this challenge, we interacted with the BREAST-Q team after the back translation to adequately understand the intent of each of the words. Thereafter, the translation team coined descriptive phrases and sentences which conveyed the intended meanings of the words. These were further refined during the process of cognitive debriefing when participants suggested further simplification of some of the expressions.

Assessment of the psychometric properties of the translated tool showed an excellent performance in most domains in terms of internal consistency reflecting the coherence of the components of the scale. Test-retest reliability which assessed the stability of the scale showed moderate reliability in most domains. Our results clearly show that the most stable concepts were those relating to physical symptoms such as pain and discomfort, which were not expected to vary significantly at the time of the interview which was at least 6 months after mastectomy. Psychological and sexual concepts which are relatively more dynamic issues however showed some variability, thus the lower scores. Test-retest reliability requires two administrations under the assumption that no change in the concept of interest has occurred. Although a time frame of 6 months to year was chosen as the reference point in this study, it is not known if women would have fully stabilized in all domains of their health by this time. The variability so observed might therefore be a reflection of a true change in these concepts based on the evolving experiences of the respondents. This assumption is substantiated by the similar pattern obtained with the already validated EORTCQLQ-BR23 tool. This study showed a strong correlation between BREAST-Q and the EORTCQLQ-BR23 in most of the domains. The best correlations were observed between the BREAST-Q sexual wellbeing and EORTCQLQ-BR23 sexual functioning, BREAST-Q psychosocial wellbeing and EORTCQLQ-BR23 body image, BREAST-Q physical wellbeing and EORTCQLQ-BR23 breast symptoms, and the BREAST-Q physical wellbeing and EORTCQLQ-BR23 arm symptoms. The correlation between BREAST-Q satisfaction and EORTCQLQ-BR23 body image domains was however weak. The weak correlation observed is not surprising, given that the two domains appear to measure slightly different constructs. The lack of a satisfaction domain in the EORTCQLQ-BR23 questionnaire necessitated the choice of the body image domain for the assessment.

Satisfaction with care was difficult to assess in this study because of the observed ceiling effect. The fact that the questionnaire was administered by a hospital staff might have influenced the responses of the participants. A similar observation was made in the Japanese translation of the BREAST-Q in which the questionnaires were administered from the same hospital where the patients received care [25]. It is possible to have obtained a different result if the surveys were administered by an independent group.

Nigeria is a multilinguistic country with over two hundred million people with different cultures and languages. There are several languages in Nigeria, Yoruba, Hausa and Igbo being the most common. Although English is the official language, there are many citizens who can only communicate in their local dialect. There are about 50 million native Yoruba speakers, the majority in the South-western part of the country. The availability of the BREAST-Q tool in a local, native dialect will ensure that the experiences of women who cannot communicate or comprehend in English language can be fully captured and given necessary attention. The lessons learnt during this process will be utilized in translating the BREAST-Q into Hausa which is the major language in the North and Igbo which is the indigenous language in the South-Eastern part of Nigeria. It is noteworthy that the Yoruba version of the BREAST-Q might also have some applicability beyond Nigeria given the presence of established Yoruba communities in some parts of the world such as Brazil, Cuba, Sierra Leone, Benin Republic and Togo, with additional cultural tailoring [26]. The availability of this tool in an indigenous language provides a unique opportunity to adequately capture the experiences of affected women. This will provide a rational basis for developing contextually relevant interventions capable of improving the quality of life of Nigerian women who have undergone mastectomy.

Overall, this study has provided an objective tool for assessing the psychosocial experiences of Yoruba-speaking Nigerian women, who have undergone mastectomy, particularly in the south-Western part of Nigeria. The translation of this PROM tool into an indigenous Nigerian language is one of the initial steps in addressing the unmet psychosocial needs of many Nigerian women who undergo mastectomy. This tool is currently being utilized in a multi-institutional longitudinal study which is comprehensively evaluating the psychosocial needs of Nigerian women and the changes they experience over time following mastectomy.

## Conclusions

The Yoruba version of the BREAST-Q mastectomy module provides a unique opportunity to adequately capture the experiences of women who undergo mastectomy for breast cancer in Nigeria. The goal of this line of research is to understand the unmet needs of breast cancer patients in Nigeria and ultimately design targeted interventions to address them.

## Data Availability

Data will be made available on reasonable request.
